# CstF-64 and 3′-UTR cis-element determine Star-PAP specificity for target mRNA selection by excluding PAPα

**DOI:** 10.1093/nar/gky1248

**Published:** 2018-12-19

**Authors:** Divya T Kandala, Nimmy Mohan, Vivekanand A, Sudheesh AP, Reshmi G, Rakesh S Laishram

**Affiliations:** 1 Cancer Research Program, Rajiv Gandhi Centre for Biotechnology, Trivandrum 695014, India; 2 Manipal Academy of Higher Education, Manipal 576104, India

*Nucleic Acids Research*, 2016, Vol. 44, No. 2, Pages 811–823, https://doi.org/10.1093/nar/gkv1074

The authors Nimmy Mohan and Sudheesh AP have added additional affiliation details:

Manipal Academy of Higher Education, Manipal 576104, India.

